# Digital phenotyping and passive monitoring as tools for personalized treatment of bipolar disorder – a literature review

**DOI:** 10.1192/j.eurpsy.2026.10710

**Published:** 2026-07-31

**Authors:** M. Bibik, A. K. Sikora

**Affiliations:** Mazovian Regional Hospital “Drewnica”, Ząbki, Poland

## Abstract

**Introduction:**

Bipolar disorder (BD) is a chronic and recurrent mental illness affecting over 40 million people globally and 1–2% of the adult population in Poland (WHO, 2019; Wolańczyk, 2014; Czerska, 2018). It is marked by a high relapse rate (39–52% annually) and significant clinical and social burden (Giménez-Palomo et al., 2024). Recognizing prodromal symptoms is crucial for preventing relapses, yet requires highly sensitive and accessible tools (Basquin et al., 2024). The development of mobile technologies and digital methods opens new avenues in psychiatry, including digital phenotyping and passive behavioral monitoring.

**Objectives:**

This review aimed to explore the use of digital tools—mobile apps, sensors, and passive monitoring systems—in predicting relapses and personalizing treatment for patients with BD.

**Methods:**

A literature review was conducted across PubMed, Scopus, and Web of Science (2015–2025), including original, randomized, pilot, and systematic studies focused on digital phenotyping, mobile applications, and passive monitoring (e.g., activity, sleep, circadian rhythms, heart rate variability, GPS, and smartphone data).

**Results:**

The review revealed growing interest in digital psychiatry. For example, the KIOS-Bipolar app (Elsayed et al., 2024) improved depression, mania, and emotional instability scores with high system acceptability. The MONARCA II system improved quality of life and reduced manic episodes but had no effect on relapse rates (Faurholt-Jepsen et al., 2020). Other solutions include MoodRhythm (circadian rhythm/sleep tracking), MoodScope (smartphone use patterns for mood prediction), BiAffect (typing dynamics), Mindstrong (digital cognitive biomarkers), and the Beiwe Research Platform(passive behavioral monitoring).

However, findings remain inconclusive. Not all apps reduce relapse rates. Key limitations include methodological inconsistencies, small samples, short follow-up, and ethical/privacy concerns.

**Conclusions:**

Digital phenotyping and passive monitoring offer promising, yet still evolving tools in psychiatry. They may support personalized care and early relapse detection, but current solutions face limitations in effectiveness, validation, and clinical integration. Future multicenter studies are needed to develop practical, ethical, and patient-centered tools for BD care.

**Disclosure of Interest:**

None Declared

